# The vaccination status of COVID-19 hospitalized patients during the Omicron BQ.1.1 wave in Northeast Brazil suggests the need for a fifth booster dose in the elderly, with a time since the last dose of more than 6 months

**DOI:** 10.17179/excli2023-5807

**Published:** 2023-02-01

**Authors:** Taise Ferreira Cavalcante, Waneska de Souza Barboza, Paulo Ricardo Martins-Filho

**Affiliations:** 1 Investigative Pathology Laboratory, Federal University of Sergipe, Aracaju, Brazil; 2 Graduate Program in Health Sciences, Federal University of Sergipe, Aracaju, Brazil; 3 Aracaju City Hall, Municipal Health Department, Aracaju, Brazil

## ⁯

Vaccines against COVID-19 have been shown to be the most effective, safe, and cost-effective measure in preventing hospitalizations, admissions to intensive care units (ICU), and deaths from the disease. Despite studies demonstrating that vaccinated individuals maintain T cell immunity to SARS-CoV-2 (Keeton et al., 2022[6]), it has been suggested that the virus's neutralizing activity declines over time and the need for booster doses especially against Omicron subvariants (Renia et al., 2022[10]). Furthermore, real-world studies have revealed evidence of waning vaccine effectiveness in a few months following the third (Goh et al., 2022[4]) and fourth doses (Nordström et al., 2022[9]), as well as the possibility of at least one COVID-19 wave recurrence in the range 3-9 months (Cappi et al., 2022[1]), eliciting debate on the need for additional doses at regular intervals. 

Although 80 % of the Brazilian population is immunized against COVID-19 with two doses of CoronaVac (Sinovac/Butantan), Covishield (Oxford-AstraZeneca/Fiocruz), or ComiRNAty (Pfizer/BioNTech) vaccines, or a single-dose J&J/Janssen vaccine, and at least 50 % having received a booster dose, a fifth wave of COVID-19 associated with the Omicron BQ.1.1 circulation has occurred in the country at the end of 2022, increasing the number of hospitalizations and deaths from the disease. In this retrospective cohort study, we evaluated the vaccination status among COVID-19 hospitalized patients during the Omicron BQ.1.1 wave in Brazil. The study was conducted in Aracaju, a port city and the capital of Sergipe state in the Northeast Brazil, recognized as the poorest region in the country. 

Aracaju has an area of 182.2 km^2^, an estimated population at over 600 000 inhabitants, and is divided administratively into 42 neighborhoods. In addition, more than one-third of families are low-income, and the Human Development Index is 0.770 (Brazilian Institute for Geography and Statistics; www.ibge.gov). The first case of COVID-19 in the city was confirmed on March 14, 2020, and 168 799 cases and 2606 deaths were reported until December 31, 2022. The last 10 epidemiological weeks of 2022 (from October 23 to December 31) were characterized by a sudden increase in the number of disease cases as a result of community transmission of the Omicron BQ.1.1, and during this fifth wave of COVID-19, 9332 cases and 14 deaths were confirmed in the city. In Aracaju, the COVID-19 vaccination campaign started on January 19, 2021. As of December 31, 2022, general population vaccination coverage was approximately 83 %, with 67 % having received at least one booster dose (https://todoscontraocorona.net.br/; https://transparencia.aracaju.se.gov.br/prefeitura/covid19/).

This study included all patients over the age of 18 living in Aracaju who had COVID-19 confirmed by polymerase chain reaction testing and were admitted to public and private hospitals in the city from October 23 to December 31, 2022. Data on COVID-19 were extracted from the microdata catalog of the Aracaju Municipal Health Department and included: (1) sex and age distribution; (2) pre-existing clinical conditions associated with COVID-19 disease severity (hypertension, diabetes, lung disease, malignancies, kidney disease, and heart disease); (3) date of hospital admission; (4) bed type (clinical bed or ICU); (5) vaccination status (unvaccinated, vaccinated with only one dose of vaccine, vaccinated with two doses, vaccinated with three doses, and vaccinated with four doses); (6) date of last dose received; and (7) time interval between the last dose and hospital admission. Data were reported descriptively, and an interaction analysis between age, the time interval between the last vaccine dose and hospital admission, and bed type was performed using a two-way analysis of variance (ANOVA). P-values < 0.05 were considered statistically significant. Analyses were performed using JASP software version 0.13 (JASP, Amsterdam, the Netherlands). Formal ethical approval is not required as all data included are anonymous secondary data. 

From October 23 to December 31, 2022, 147 adults were hospitalized due to COVID-19, with 69 (47 %) being male and 105 (71.4 %) having pre-existing clinical conditions associated with the COVID-19 disease severity. The median age was 77 years (Q1 = 59; Q3 = 85), and approximately 67 % of hospitalized patients were over 70 years of age (Supplementary Figure 1). A total of 110 (74.8 %) patients were admitted to clinical hospital beds and 37 (25.2 %) were admitted to an ICU. During the study period, the overall hospitalization rate was 1.6 %. Regarding COVID-19 vaccine status, eight (5.4 %) patients were unvaccinated; four (2.7 %) patients had only received one dose of vaccine; and 135 (91.8 %) patients had received the primary schedule against COVID-19 with a two-dose vaccination regimen. Among those who had vaccinated with two doses, 128 (94.8 %) had received a booster (third dose) of the COVID-19 vaccine; 83 (64.8 %) had also received a fourth dose. Approximately 83 % of fully vaccinated patients with four doses were aged 70 years or older, and at least 75 % had their last dose of the COVID-19 vaccine 6 months before hospital admission (median 7.4 months; Q1 = 6.2; Q3 = 8.0) (Supplementary Figure 2). Furthermore, we found a relationship between advanced age, a time interval of at least 6 months between the last vaccine dose and hospital admission, and the need for ICU care (p = 0.017) (Supplementary Figure 3).

Consistent evidence in the literature shows that the primary COVID-19 vaccine cycle and booster doses are effective in preventing COVID-19-related outcomes in the short-term (Muhsen et al., 2022[8]). Previously, we demonstrated a decrease in hospitalization rates in this region as vaccination coverage increased, ranging from 6.9 % during the predominance of the Gamma and Zeta variants (January - June 2021; vaccination coverage: 12 %) to 1.7 % during the wave associated with the Omicron variant (January - February 2022; vaccination coverage: 73 %) (Martins-Filho et al., 2022[7]). The current study showed a hospitalization rate comparable to that observed in early 2022, emphasizing the importance of the COVID-19 vaccination campaign as new Omicron subvariants emerge. 

However, studies have found a progressive decline in the humoral response to SARS-CoV-2 during the first 6 months after infection (de Lima Silva et al., 2022[2]) or after being fully vaccinated (Rodriguez et al., 2022[11]). A systematic review with meta-regression analysis showed a reduction in vaccine effectiveness of 32.0 and 9.5 percentage points for symptomatic and severe COVID-19 disease, respectively, in older people 6 months after vaccination (Feikin et al., 2022[3]). Furthermore, there is evidence of loss of antibodies and reduced vaccine effectiveness in the first 6 months after the booster doses. A test-negative study (Grewal et al., 2022[5]) conducted among long-term care residents aged ≥ 60 years in Ontario, Canada, found that the fourth dose provided additional protection against Omicron-related outcomes, but the protection waned over time. The authors suggested an additional booster 4-6 months after fourth dose receipt to continue to provide increased protection against SARS-CoV-2 infection for this vulnerable population. 

Almost 70 % of hospitalized patients in the current study were over 70 years old, and slightly more than half had received four doses of the vaccine. Furthermore, among those fully vaccinated with all four doses, we found a greater need for ICU care when the interval between the last dose and the date of admission was longer than 6 months. These findings suggest that the fourth dose of COVID-19 vaccine has a short-term protective effect in preventing more severe disease cases. Therefore, the vaccination status of COVID-19 hospitalized patients during the Omicron BQ.1.1 wave in Northeast Brazil suggests the need of public policies to complete the vaccination schedule with four doses in the adult population. Despite the limitations of this study, including the small sample size and the lack of longitudinal data of neutralizing antibody titers, our findings may also suggest the need for a fifth dose among those over 70 years, with a time since the last dose of more than 6 months.

## Declaration

### Authors' contributions

All authors contributed equally to this work.

### Conflict of interest statement

The authors have no conflicts of interest to declare.

### Role of funding source

There is no funding source.

## Supplementary Material

Supplementary information

## References

[1] Cappi R, Casini L, Tosi D, Roccetti M (2022). Questioning the seasonality of SARS-COV-2: a Fourier spectral analysis. BMJ Open.

[2] de Lima Silva N, Nobre D, Alves de Oliveira J, Santos Rezende M, da Conceição Dos Santos JT, de Souza Araújo AA (2022). Kinetics of humoral immune response in patients with asymptomatic or mild COVID-19: a longitudinal study based in an in-house indirect ELISA method. EXCLI J.

[3] Feikin DR, Higdon MM, Abu-Raddad LJ, Andrews N, Araos R, Goldberg Y (2022). Duration of effectiveness of vaccines against SARS-CoV-2 infection and COVID-19 disease: results of a systematic review and meta-regression. Lancet.

[4] Goh YS, Rouers A, Fong S-W, Zhuo NZ, Hor PX, Loh CY (2022). Waning of specific antibodies against Delta and Omicron variants five months after a third dose of BNT162b2 SARS-CoV-2 vaccine in elderly individuals. Front Immunol.

[5] Grewal R, Nguyen L, Buchan SA, Wilson SE, Costa AP, Kwong JC (2022). Effectiveness and duration of protection of a fourth dose of coronavirus disease 2019 messenger RNA vaccine among long-term care residents in Ontario, Canada. J Infect Dis.

[6] Keeton R, Tincho MB, Ngomti A, Baguma R, Benede N, Suzuki A (2022). T cell responses to SARS-CoV-2 spike cross-recognize Omicron. Nature.

[7] Martins-Filho PR, de Souza Araújo AA, Quintans-Júnior LJ, Soares B dos S, Barboza W de S, Cavalcante TF (2022). Dynamics of hospitalizations and in-hospital deaths from COVID-19 in northeast Brazil: a retrospective analysis based on the circulation of SARS-CoV-2 variants and vaccination coverage. Epidemiol Health.

[8] Muhsen K, Maimon N, Mizrahi AY, Boltyansky B, Bodenheimer O, Diamant ZH (2022). Association of receipt of the fourth BNT162b2 dose with Omicron infection and COVID-19 hospitalizations among residents of long-term care facilities. JAMA Intern Med.

[9] Nordström P, Ballin M, Nordström A (2022). Effectiveness of a fourth dose of mRNA COVID-19 vaccine against all-cause mortality in long-term care facility residents and in the oldest old: A nationwide, retrospective cohort study in Sweden. Lancet Reg Heal Eur.

[10] Renia L, Goh YS, Rouers A, Le Bert N, Chia WN, Chavatte J-M (2022). Lower vaccine-acquired immunity in the elderly population following two-dose BNT162b2 vaccination is alleviated by a third vaccine dose. Nat Commun.

[11] Rodriguez PE, Silva AP, Miglietta EA, Rall P, Pascuale CA, Ballejo C (2022). Humoral response and neutralising capacity at 6 months post-vaccination against COVID-19 among institutionalised older adults in Argentina. Front Immunol.

