# Supplementary material for: The vaccination status of COVID-19 hospitalized patients during the Omicron BQ.1.1 wave in Northeast Brazil suggests the need for a fifth booster dose in the elderly, with a time since the last dose of more than 6 months
Source: EXCLI J. 2023 Feb 1;22:169–72. doi: 10.17179/excli2023-5807 (PMC10043451; doi:10.17179/excli2023-5807)
Supplement: Supplementary information [file EXCLI-22-169-s-001.pdf]

EXCLI Journal 2023;22:169-172 – ISSN 1611-2156
Received: January 10, 2023, accepted: January 31, 2023, published: February 01, 2023

Supplementary information to:

Letter to the editor:

THE VACCINATION STATUS OF COVID-19 HOSPITALIZED PATIENTS DURING THE OMICRON BQ.1.1 WAVE IN NORTHEAST BRAZIL SUGGESTS THE NEED FOR A FIFTH BOOSTER DOSE IN THE ELDERLY, WITH A TIME SINCE THE LAST DOSE OF MORE THAN 6 MONTHS

Taise Ferreira Cavalcante[1,2,3], Waneska de Souza Barboza[3],
Paulo Ricardo Martins-Filho[1,2]

1 Investigative Pathology Laboratory, Federal University of Sergipe, Aracaju, Brazil
2 Graduate Program in Health Sciences, Federal University of Sergipe, Aracaju, Brazil
3 Aracaju City Hall, Municipal Health Department, Aracaju, Brazil

* **Corresponding author:** Prof. Paulo Ricardo Martins-Filho. Universidade Federal de Sergipe, Hospital Universitário, Laboratório de Patologia Investigativa. Rua Cláudio Batista, s/n. Sanatório. Aracaju, Sergipe, Brasil. CEP: 49060-100.
E-mail: prmartinsfh@gmail.com

https://dx.doi.org/10.17179/excli2023-5807

This is an Open Access article distributed under the terms of the Creative Commons Attribution License (http://creativecommons.org/licenses/by/4.0/).

EXCLI Journal 2023;22:169-172 – ISSN 1611-2156
Received: January 10, 2023, accepted: January 31, 2023, published: February 01, 2023

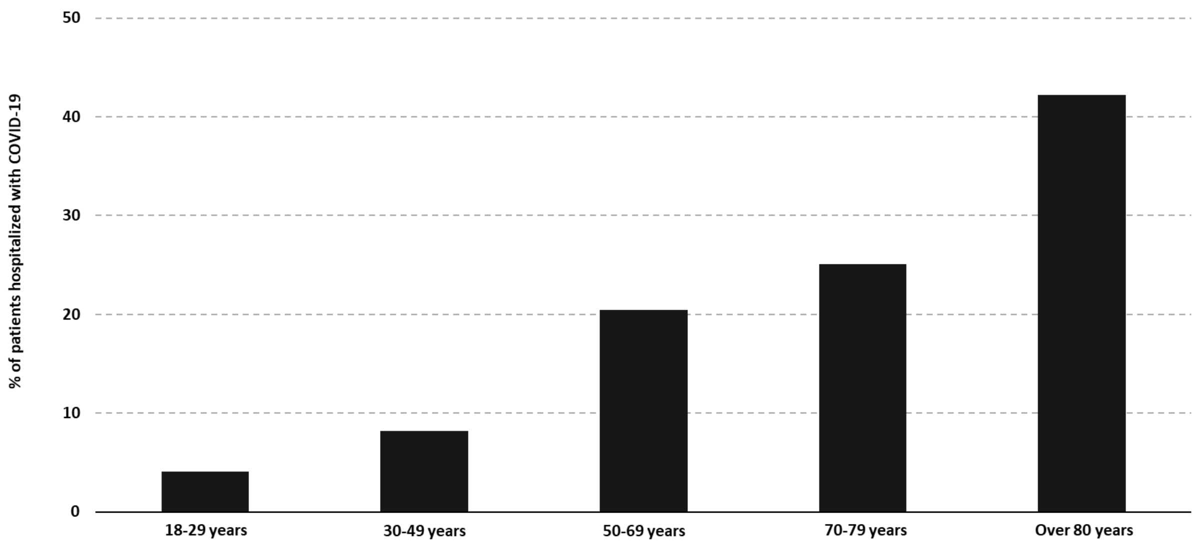

Supplementary Figure 1: Percentage of COVID-19 hospitalized patients according to age

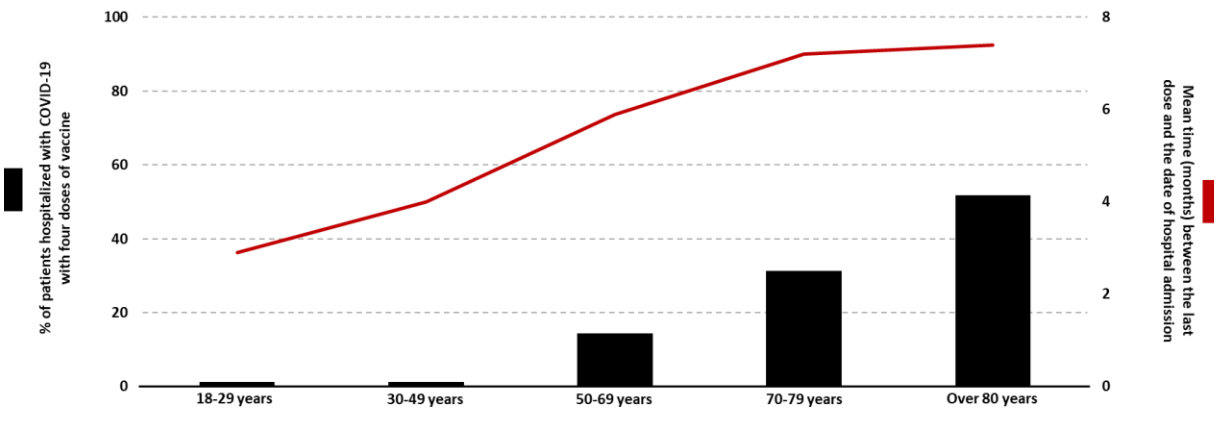

Supplementary Figure 2: Percentage of patients hospitalized with COVID-19 who received the fourth dose of the vaccine and the mean time between the last dose and the date of admission

EXCLI Journal 2023;22:169-172 – ISSN 1611-2156
Received: January 10, 2023, accepted: January 31, 2023, published: February 01, 2023

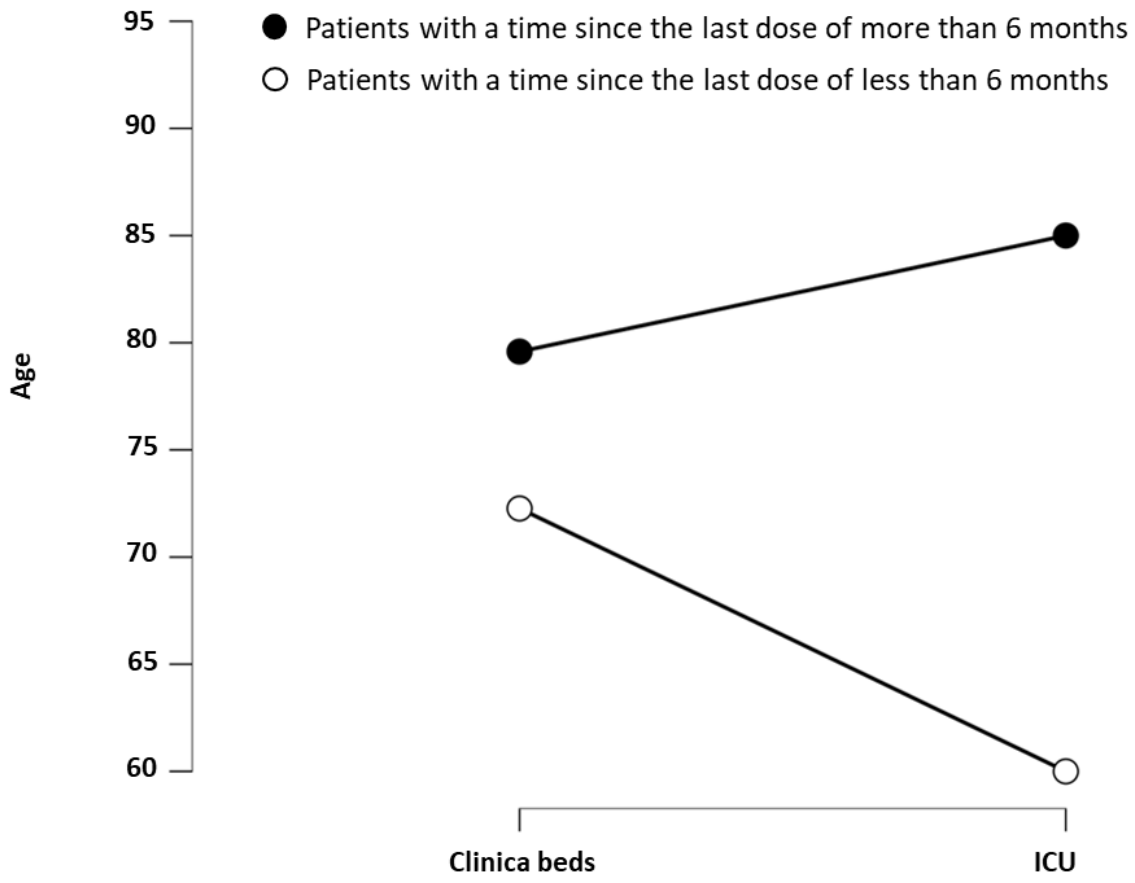

Supplementary Figure 3: Interaction analysis between age, the time interval between the last vaccine dose and hospital admission, and bed type
